# Long-term multidimensional patient-centred outcomes after hospitalisation for COVID-19: do not only focus on disease severity

**DOI:** 10.1136/bmjresp-2024-002789

**Published:** 2025-06-08

**Authors:** Martine Bek, Yasemin Türk, Matthijs L Janssen, Gemma Weijsters, Julia C Berentschot, Rita J G van den Berg-Emons, Majanka H Heijenbrok-Kal, Gerard M Ribbers, Joachim Aerts, Wessel E J J Hanselaar, Henrik Endeman, Merel E Hellemons, Evert-Jan Wils, CO-FLOW collaboration group, Joachim GJV Aerts, Dutch HFNO COVID-19 study group, Yaar Aga

**Affiliations:** 1 Department of Rehabilitation Medicine, Erasmus MC, University Medical Center Rotterdam, Rotterdam, The Netherlands; 2 Department of Respiratory Medicine, Franciscus Gasthuis & Vlietland Hospital, Rotterdam, The Netherlands; 3 Department of Intensive Care, Franciscus Gasthuis & Vlietland Hospital, Rotterdam, The Netherlands; 4 Department of Intensive Care, Erasmus MC, University Medical Center Rotterdam, Rotterdam, The Netherlands; 5 Department of Respiratory Medicine, Erasmus MC, University Medical Center Rotterdam, Rotterdam, The Netherlands; 6 Rijndam Rehabilitation, Rotterdam, The Netherlands

**Keywords:** COVID-19, Respiratory Infection, Critical Care, Patient Outcome Assessment

## Abstract

**Objectives:**

To investigate the association between COVID-19 disease severity during hospitalisation for COVID-19 and long-term multidimensional patient-centred outcomes up to 12 months post-hospitalisation. The secondary objective was to identify other risk factors for these long-term outcomes.

**Methods:**

In this multicentre prospective cohort study, we categorised COVID-19 disease severity using the maximal level of respiratory support as proxy into (1) conventional oxygen therapy (COT), (2) high-flow nasal oxygen (HFNO) and (3) invasive mechanical ventilation (IMV). The primary outcome health-related quality of life (HRQoL), and the secondary outcomes self-reported symptoms and recovery were collected at 6 and 12 months post-hospitalisation.

**Results:**

Data from 777 patients were analysed, with 226 (29%) receiving COT, 273 (35%) HFNO and 278 (36%) IMV. Patients reported impaired HRQoL, persistence of symptoms and poor recovery. Multivariable generalised estimating equations analysis showed that COVID-19 disease severity was not associated with HRQoL and inconsistently with symptoms; the HFNO group reported poorer recovery. Overall, female sex, younger age and pulmonary history were independent risk factors for outcomes.

**Conclusions:**

COVID-19 disease severity was associated with self-perceived recovery, but not with HRQoL and inconsistently with symptoms. Our findings suggest that age, sex and pulmonary history are more consistent risk factors for long-term multidimensional outcomes and offer better guidance for aftercare strategies.

**WHAT IS ALREADY KNOWN ON THIS TOPIC**

- COVID-19 often leads to hypoxaemia and respiratory failure, necessitating hospitalisation and varying levels of oxygen therapy. Previous studies have shown that the severity of the initial illness can influence these outcomes, but the specific impact of different levels of respiratory support on long-term patient-centred outcomes remains unclear.

**WHAT THIS STUDY ADDS**

- This study demonstrates that COVID-19 disease severity, defined by the maximal level of respiratory support, is associated with perceived recovery, but not consistently with health-related quality of life or symptom persistence. Instead, it identifies female sex, younger age and pulmonary history as more reliable predictors of long-term health outcomes than the severity of the acute illness alone.

**HOW THIS STUDY MIGHT AFFECT RESEARCH, PRACTICE OR POLICY**

- Our findings collectively suggest that aftercare strategies for patients with COVID-19 should prioritise individual risk factors like age, sex and pulmonary history over disease severity alone. This approach may enhance the effectiveness of post-hospitalisation care and improve long-term outcomes for patients.

## Introduction

 Hypoxaemia and respiratory failure are the primary reasons for hospitalisation in patients with COVID-19.^1^ Depending on the severity of hypoxaemia, oxygen therapy can range from low-flow nasal oxygen or oxygen masks to non-invasive respiratory support modalities (high-flow nasal oxygen (HFNO), non-invasive mechanical ventilation (NIV)) or even invasive mechanical ventilation (IMV).^2^

After hospitalisation, patients frequently report long-term health problems,^3 4^ known as post-COVID-19 condition or ‘long covid’.^5^ These problems include a reduced health-related quality of life (HRQoL), persistence of symptoms and incomplete self-perceived recovery.^4 6 7^ Half of the patients with mild disease and up to 90% of people with moderate and severe disease continue to experience at least one ongoing symptom 1 year after disease onset.^45 7^,^9^ Mild disease often refers to non-hospitalised cases, whereas hospitalised patients are typically classified as having moderate to severe disease. Hospitalised patients requiring oxygen more frequently suffer from a reduced HRQoL and from symptoms compared with those with milder disease severity and those without hospitalisation.^46 7 10^,^12^ However, the association between COVID-19 disease severity and long-term outcomes in hospitalised patients requiring oxygen is uncertain. A more granular understanding of this association may help to guide, prioritise and optimise treatment and aftercare strategies.

We hypothesised that long-term patient-centred outcomes are related to disease severity. Therefore, we assessed the association between the maximal level of respiratory support received during hospitalisation, as a proxy for COVID-19 disease severity, and long-term multidimensional patient-centred outcomes (HRQoL, symptoms and recovery) up to 12 months in patients hospitalised for COVID-19 requiring oxygen therapy. We further aimed to identify other risk factors for these long-term outcomes.

## Material and methods

### Study design

Data from two multicentre prospective cohort studies conducted at 13 healthcare institutions in the Netherlands were combined: The Dutch HFNO COVID-19 study with trial register no. NL9067 and the COVID-19 Follow-up care paths and Long-term Outcomes Within the Dutch healthcare system (CO-FLOW) study with trial register no. NL8710 (both registered on the WHO ICTRP). The study has been carried out in accordance with the Declaration of Helsinki for medical research involving humans. Details of the HFNO COVID-19 study and the CO-FLOW study have been described previously.^13 14^ We reported this observational study according to Strengthening the Reporting of Observational Studies in Epidemiology (STROBE) guidelines; the STROBE checklist is provided in the online supplemental material.

### Participants

Eligible participants were hospitalised adults with confirmed COVID-19 infection who received treatment with conventional oxygen therapy (COT), HFNO and/or IMV, and survived to hospital discharge. We excluded patients treated with NIV (n=18) as maximal level of respiratory support, as NIV was rarely applied in participating centres. All participants provided informed consent before data collection.

### Data collection

Demographics and clinical characteristics were collected in both cohorts according to their respective study protocols previously^13 14^ (online supplemental table S1). Data were collected during study visits, from medical records and via patient-reported outcomes measures sent via mail or postal mail.

Participants of the HFNO COVID-19 study admitted between 1 December 2020 and 31 March 2021 received questionnaires only 12 months after discharge, as the 6-month follow-up had already passed at the time of inclusion for this follow-up study. Participants admitted between 1 April 2021 and 30 June 2021 received questionnaires 6 months after discharge. Participants of the CO-FLOW study included between 1 July 2020 and 1 September 2021 received questionnaires at 6 and 12 months after hospital discharge. Data were stored in the Castor Electronic Data Capture System (Castor EDC, Amsterdam, The Netherlands).

### Outcomes

The primary outcome was HRQoL as measured with the EuroQol Group 5 Level, 5 Dimension descriptive system (EQ-5D-5L), consisting of the EQ-5D descriptive system and the EQ Visual Analogue Scale (EQ-VAS).^15^ The EQ-5D-5L utility score was calculated according to the Dutch tariff for the EQ-5D-5L ranging from 0 (death) to 1 (best health possible). The mean (SD) Dutch reference value for the general population is 0.87 (0.17) and median value (IQR) is 0.89 (0.82–1.00).^16^ The EQ-VAS records the participant’s self-rated health through a VAS ranging from 0 (worst imaginable health) to 100 (best health), to assess subjective general health. The mean (SD) Dutch reference value is 80.6 (14.7) and median value (IQR) is 81.0 (72.0–90.0).

Secondary outcomes were self-reported COVID-19 symptoms and self-reported recovery status. Symptoms were measured with the Corona Symptom Checklist that has been developed within the CO-FLOW study to assess newly developed or worsened symptoms since the onset of COVID-19 with ‘yes’ or ‘no’ as answer options.^13 17^ Recovery status was assessed via the Core Outcome Measure for Recovery of COVID-19 using a 5-point Likert scale ranging from ‘not recovery at all’ to ‘completely recovered’.^18^

### Data analysis

Data were analysed from participants from whom data on the primary outcome of interest was available at one of the follow-up moments. Data were presented as mean (SD) and/or median (IQR), as applicable, for continuous variables and as number with percentage (%) for categorical variables. Participants were categorised into three groups according to their maximal level of respiratory support received during hospitalisation, in line with the WHO clinical progression scale: ≤15 L/min of (COT group, WHO score 5); (HFNO group, WHO score 6) and (IMV group, WHO score 7–9). We used the Kruskal-Wallis test and χ^2^ test for group comparisons.

We used univariable linear generalised estimating equations (GEEs) to investigate the association between maximal level of respiratory support and HRQoL, that is, EQ-5D-5L utility and EQ-VAS score at 6 and 12 months, respectively. Second, we used multivariable linear GEE analysis with clinically important characteristics^4 19^ (online supplemental table S1) to identify additional risk factors for HRQoL. A priori, we had determined that the maximal level of respiratory support should be kept in all models irrespective of statistical significance. GEE analysis was used because it employs quasi-likelihood estimation and robust (sandwich) variance estimators, making it suitable for both linear and categorical outcomes in cross-sectional analyses, particularly when the data are not normally distributed.

We categorised self-reported symptoms into four clusters: physical, respiratory, fatigue and cognitive symptom cluster as done previously.^17^ Self-reported recovery was classified into good recovery (mostly and completely recovered) and poor recovery (not, somewhat and half recovered).

Similarly, binary GEE analyses were performed for persistence of symptoms and recovery. We did an exploratory analysis with the duration of HFNO treatment and respiratory and fatigue symptoms and recovery. A p<0.05 was considered statistically significant. Models are presented as forest plots with adjusted β or adjusted ORs (AORs), 95% CI and p values. Analyses were performed using SPSS Statistics V.28 (IBM SPSS Statistics, SPSS).

### Patient and public involvement

Patients were not directly involved in the design of the studies. However, for the CO-FLOW study, patients were involved in the development of a patient-reported experience questionnaire on satisfaction with COVID-19 aftercare, which we assessed 1 year after hospital discharge (not included in this analysis), and patient input was used in the continuation of the CO-FLOW study. The study was implemented during the early stages of the pandemic, and any patient and public involvement could have delayed its rapid implementation. We disseminate the main results of the study to the study participants and the wider public via newsletters, social media and conferences.

## Results

### Cohort characteristics

A total of 1036 participants were invited for long-term follow-up evaluation between January 2021 and April 2022, 725 patients at 6 months and 854 at 12 months; 555 participants from the CO-FLOW study and 481 of the HFNO COVID-19 study (online supplemental figure S1), respectively. A total of 295 patients were excluded for the current study. At 6 months, 497 (68.6%) participants completed the EQ-5D-5L questionnaire and 620 (72.6%) participants at 12 months. Patient characteristics (body mass index, respiratory support, smoking status, intensive care unit (ICU) admission and hospital length of stay) differed between non-responders and responders at 6 months, but not at 12 months (online supplemental table S2). Clinically relevant differences in baseline characteristics were not present between the participants evaluated at 6 and 12 months (online supplemental table S3). The current analysis included 777 participants in whom the primary outcome was available at one of the follow-up moments. Of those, 226 (29.1%) received COT, 273 (35.1%) HFNO and 278 (35.8%) IMV as maximal level of respiratory support. Detailed characteristics of participants are shown in table 1.

**Table 1:** Baseline characteristics of the full cohort and split by the maximal level of respiratory support

	Full cohort	COT	HFNO	IMV	P value
N	777	226 (29.1)	273 (35.1)	278 (35.8)	
Demographics					
Sex, female	241 (31.0)	80 (35.4)	92 (33.7)	69 (24.8)	0.02
Age at admission, years	60.0 (53.0–67.0)	61.0 (54.0–68.0)	60.0 (53.0–67.0)	60.0 (54.0–67.3)	0.60
BMI at admission, kg/m²*	28.5 (25.9–32.3)	27.5 (25.2–31.0)	28.5 (25.8–32.2)	29.4 (26.6–33.5)	<0.001
Physical activity level*					0.11
Inactive	119 (15.5)	32 (14.3)	51 (18.8)	36 (13.1)	
Light	410 (53.3)	115 (51.6)	136 (50.0)	159 (58.0)	
Moderate	203 (26.4)	60 (26.9)	77 (28.3)	66 (24.1)	
Vigorous	37 (4.8)	16 (7.2)	8 (2.9)	13 (4.7)	
Smoking status, ex-/current*	355 (49.9)	126 (56.2)	97 (42.7)	132 (50.8)	0.025
Clinical characteristics					
Medical history					
≥1	589 (75.8)	163 (72.1)	202 (74.0)	224 (80.6)	0.06
Obesity (BMI≥30)	296 (39.7)	72 (31.9)	95 (39.3)	129 (46.6)	0.004
Cardiovascular disease	305 (39.3)	86 (38.1)	101 (37.0)	118 (42.4)	0.39
Pulmonary disease	181 (23.3)	52 (23.0)	68 (24.9)	61 (21.9)	0.71
Diabetes	156 (20.1)	40 (17.7)	59 (21.6)	57 (20.5)	0.55
CRP, mg/L*	103.0 (56.0–165.0)	69.0 (39.3–114.8)	112.0 (68.0–172.8)	125.0 (69.0–195.0)	<0.001
Creatinine, µmol/L*	83.0 (69.0–100.0)	79.0 (65.0–94.5)	80.0 (66.5–94.0)	88.0 (75.0–110.5)	<0.001
Pharmaceutical treatment during admission†					
No specific treatment	93 (12.0)	48 (21.2)	3 (1.1)	42 (24.6)	0.003
Antivirals	74 (9.5)	47 (20.8)	14 (5.1)	13 (4.7)	<0.001‡
Steroids	655 (84.3)	166 (73.5)	266 (97.4)	223 (80.2)	<0.001‡
Anti-inflammatory	239 (30.8)	2 (0.9)	122 (44.7)	115 (41.4)	<0.001‡
HFNO	470 (60.5)	0 (0)	273 (100)	197 (70.9)	<0.001
Duration of HFNO, days		NA	5.0 (3.0–7.0)	1.0 (1.0–3.0)	<0.001
ICU admission	380 (48.9)	6 (2.7)	96 (35.2)	278 (100)	<0.001
ICU LOS, days	11.0 (6.0–25.0)	2.0 (0.0–6.5)	3.5 (2.0–6.0)	16.0 (10.0–30.0)	<0.001
Length of IMV, days		NA	NA	12.0 (7.0–24.0)	NA
Postdischarge care*					<0.001
No or community-based rehabilitation	521 (72.9)	217 (96.0)	213 (88.4)	91 (36.7)	
Medical rehabilitation	97 (13.6)	1 (0.4)	7 (2.9)	89 (35.9)	
Skilled nursing rehabilitation	97 (13.6)	8 (3.5)	21 (8.7)	68 (27.4)	
Hospital LOS, days*	13.0 (8.0–26.0)	6.0 (4.0–10.3)	11.0 (11.0–15.0)	32.0 (20.0–46.0)	<0.001
Time interval between hospital discharge and follow-up visit					
6 months visit, months	6.0 (6.0–7.0)	6.0 (6.0–6.0)	7.0 (6.0–8.0)	7.0 (6.0–7.0)	
12 months visit, months	12.0 (12.0–13.0)	12.0 (12.0–12.0)	12.0 (11.0–12.0)	12.0 (12.0–13.0)	

Data are presented as median (IQR) or n (%). P value is obtained using Kruskal-Wallis test or χ2 test as appropriate.

* Missing values (n (%)) in BMI at admission, n=72 (9.3%); pre-COVID-19 smoking status, n=66 (8.5%); pre-COVID-19 physical activity level, n=8 (1.0%); creatinine at admission, n=18 (2.3%); CRP at admission, n=19 (2.4%); LOS hospital, n=4 (0.5%); postdischarge care, n=62 (8.0).

† Treatment strategies during hospitalisation varied between the CO-FLOW study and HFNO COVID-19 study related to the different inclusion periods. The distribution (n (%)) is as follows: CO-FLOW vs HFNO COVID-19: no treatment: n=97 (21.1%) vs n=3 (0.9%); antivirals: n=64 (13.9%) vs n=10 (3.2%); steroids: n=341 (74.1%) vs n=314 (99.1%); anti-inflammatory: n=59 (12.8%) vs n=180 (56.8%).

‡ COT groups received most often antiviral treatment, but less often anti-inflammatory treatment, HFNO group received most often treatment with steroids.

BMI, body mass index; COT, conventional oxygen therapy; CRP, C reactive protein; HFNO, high-flow nasal oxygen; ICU, intensive care unit; IMV, invasive mechanical ventilation; LOS, length of stay; NA, not applicable.

### Disease severity and HRQoL

**Figure 1:**
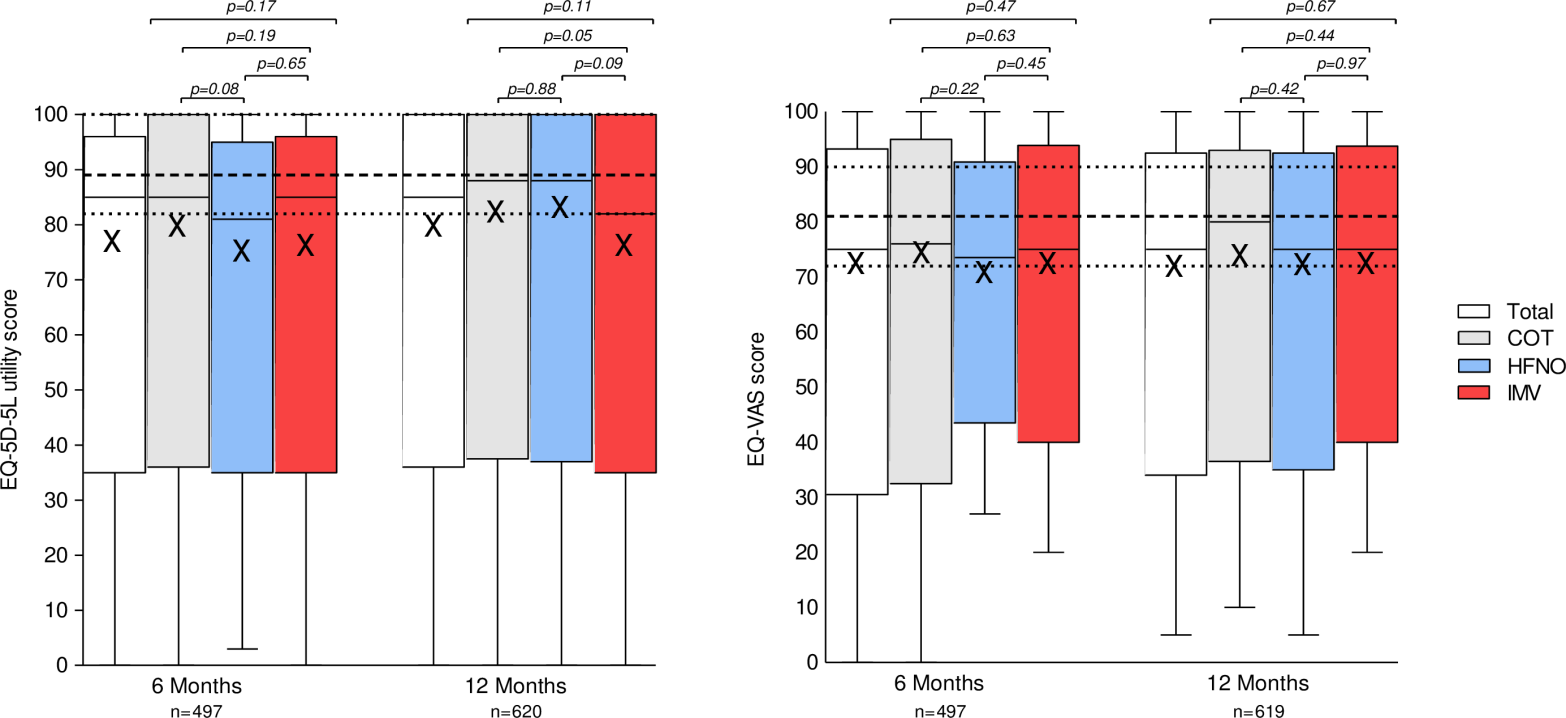
Health-related quality of life for different maximum levels of respiratory support at 6 and 12 months after hospital discharge. Boxplots showing distribution of health-related quality of life; (A) EQ-5D-5L utility score and (B) EQ-VAS at 6 and 12 months for the total cohort and for each level of respiratory support (COT, HFNO or IMV). Boxplots displaying mean, median, IQR and range. A box shows the upper and lower quartiles with the inside of the box indicating the IQR; the midline signifies the median; the whisker indicates the range with minimum and maximum values. The symbol X represents the mean. Dotted lines indicate the reference value of the Dutch population as median with IQRs. COT, conventional oxygen therapy; EQ-5D-5L, EuroQol Group 5 Level, 5 Dimension; EQ-VAS, EQ Visual Analogue Scale; HFNO, high-flow nasal oxygen; IMV, invasive mechanical ventilation.

We first assessed the association between the maximal level of respiratory support for HRQoL at 6-month and 12-month follow-up (figure 1). The EQ-5D-5L utility score did not differ significantly between the COT, HFNO and IMV groups at both 6 and 12 months. Similarly, EQ-VAS did not differ significantly between respiratory support groups at 6 and 12 months. The median EQ-5D-5L utility score was 0.85 (0.70–0.92) at 6 months and 0.85 (0.72–1.00) at 12 months (online supplemental table S4). The median EQ-VAS for the total cohort was 75.0 (61.0–86.5) at 6 months and 75.0 (63.0–85.0) at 12 months (online supplemental table S4). Both median EQ-5D-5L utility and EQ-VAS score were at 6 and 12 months significantly lower than the Dutch reference value (p<0.001). Only minor differences were observed between EQ-5D domains when stratified by respiratory support group (online supplemental table S5 and S6).

### Risk factors of HRQoL

**Figure 2:**
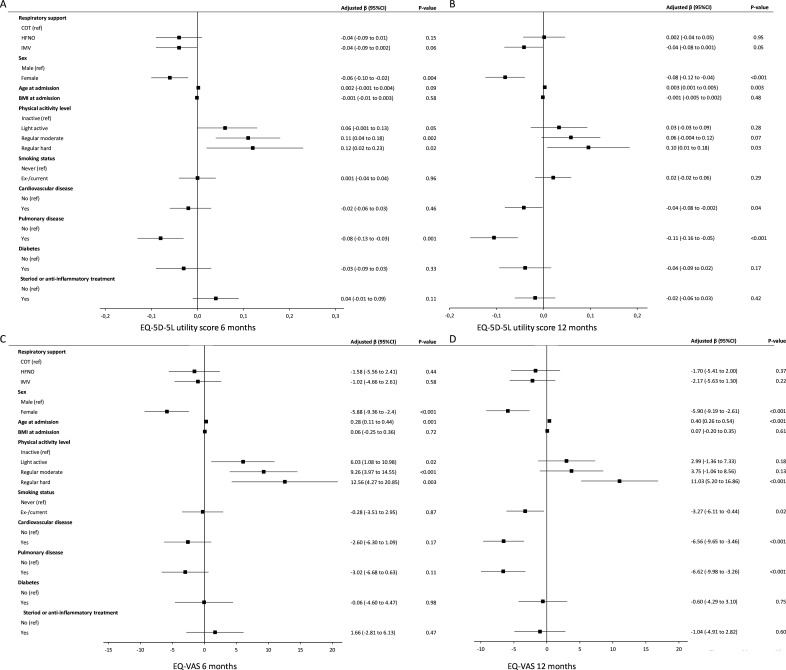
Health-related quality of life and its risk factors at 6 and 12 months after hospital discharge. Forest plots presenting risk factors for health-related quality of life (A) EQ-5D-5L utility score and (B) EQ-VAS at 6 and 12 months after hospitalisation for COVID-19. Data are obtained using multivariable linear generalised estimating equations analysis. The EQ-5D-5L utility score was calculated according to the Dutch tariff for the EQ-5D-5L ranging from 0 (death) to 1 (best health possible).^16^ The EQ-VAS ranges from 0 (worst imaginable health) to 100 (best health), to assess subjective general health. BMI, body mass index; β, beta/estimated mean; COT, conventional oxygen therapy; EQ-5D-5L, EuroQol-5 Dimension-5 Level; HFNO, high-flow nasal oxygen; IMV, invasive mechanical ventilation; VAS, visual analogue scale.

Using multivariable GEE analysis, we identified female sex, physical inactivity prior to infection and pulmonary history as risk factors for lower EQ-5D-5L utility score at 6 months (figure 2). Participants who were female, were younger, had a cardiovascular history and pulmonary history had a lower EQ-5D-5L utility score at 12 months.

Participants who were female, were younger and were physically inactive prior to infection had a lower EQ-VAS score at 6 months. These risk factors, together with an ex-/current smoking status, cardiovascular history and pulmonary history, were associated with a lower EQ-VAS score at 12 months.

### Disease severity and symptoms

At 6 months, 503 participants (64.7%) experienced symptoms in ≥1 of the symptom clusters, and at 12 months 587 (75.5%) participants. Of all symptom clusters, symptoms from the physical cluster were most prevalent at 6 and 12 months (91.0% and 87.7%, respectively) (online supplemental table S7).

We assessed the role of maximal level of respiratory support on the persistence of symptoms. The HFNO group was more likely to experience respiratory symptoms compared with the COT (AOR 2.6 (95% CI 1.6 to 4.2), p<0.001) and IMV group (2.3 (95% CI 1.4 to 3.6), p<0.001) at 6 months, but not at 12 months. At 12 months, the IMV group was at increased risk for physical symptoms compared with the COT group (2.5 (95% CI 1.4 to 4.7), p=0.003) and the HFNO group had a higher risk for fatigue symptoms compared with the COT group (1.9 (95% CI 1.3 to 2.8), p=0.002).

### Risk factors for symptoms

**Figure 3:**
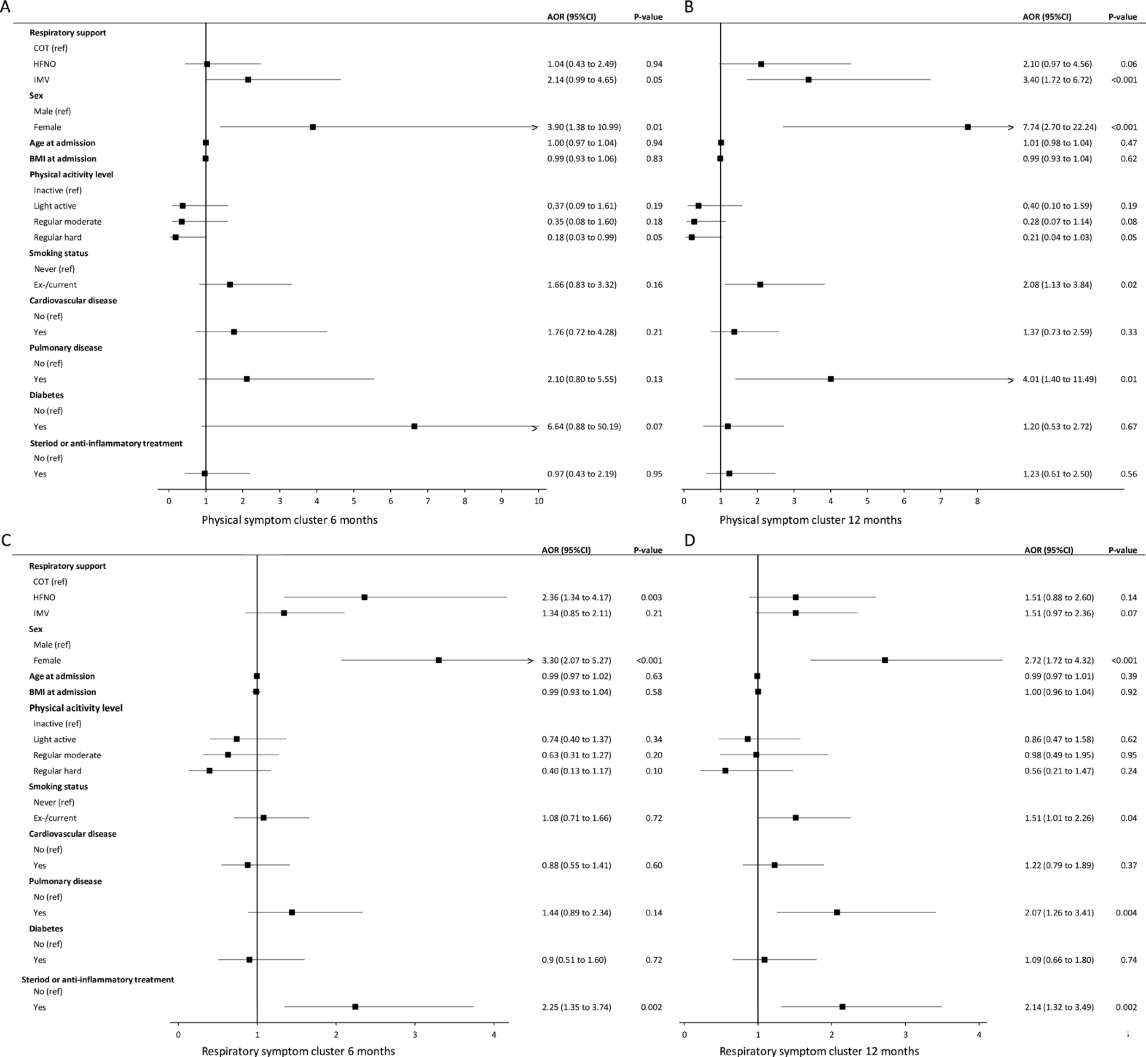
Physical and respiratory symptom cluster and their risk factors at 6 and 12 months after hospital discharge. Forest plots presenting risk factors of (A) physical symptom cluster at 6 months, (B) physical symptom cluster at 12 months, (C) respiratory symptom cluster at 6 months and (D) respiratory symptom cluster at 12 months postdischarge. Data are obtained using multivariable binary generalised estimating equations analysis. Symptoms were assessed with the Corona Symptom Checklist.^17^ AOR, adjusted OR; BMI, body mass index; COT, conventional oxygen therapy; HFNO, high-flow nasal oxygen; IMV, invasive mechanical ventilation.

We further identified potential risk factors for persistence of symptoms using multivariable GEE analysis (figure 3 and online supplemental figure S2). Female sex was the only risk factor for physical symptoms at 6 months (figure 3A). The HFNO group was more likely to experience respiratory symptoms compared with the COT (AOR 2.4 (95% CI 1.3 to 4.2), p=0.003) and IMV groups (1.8 (95% CI 1.0 to 3.1), p=0.04) (figure 3C). Other risk factors for respiratory symptoms were female sex and receiving steroid or anti-inflammatory treatment. Risk factors for fatigue symptoms were female sex, pulmonary history and receiving steroid or anti-inflammatory treatment (online supplemental figure S2A). Cognitive symptoms were associated with female sex, younger age and pulmonary history (online supplemental figure S2C).

By exploring the univariable association between HFNO treatment duration and respiratory symptoms, we observed an association in patients with HFNO as maximal level of respiratory support (n=114) (1.0 (95% CI 1.0 to 1.2), p=0.04) as well as in patients treated with HFNO and subsequent IMV (n=198) (1.1 (95% CI 1.1 to 1.2), p=0.002), at 6 months, but not at 12 months.

At 12 months, the IMV group was more likely to experience physical symptoms compared with the COT group. Other risk factors were female sex, ex-/current smoking status and pulmonary history (figure 3B). Risk factors for respiratory symptoms were female sex, ex-/current smoking status, pulmonary history and receiving steroid or anti-inflammatory treatment (figure 3D). The HFNO and IMV groups were more likely to experience fatigue symptoms compared with the COT group (online supplemental figure S2B), but there was no association with the duration of HFNO therapy. Other risk factors for respiratory symptoms were female sex, younger age and pulmonary history. Female sex was the only risk factor for cognitive symptoms (online supplemental figure S2D).

### Disease severity and recovery

At 6 months, 29% of the participants had a poor self-reported recovery and 25% at 12 months (online supplemental table S8). A higher level of maximal respiratory support was univariably associated with poorer recovery (HFNO vs COT: 2.4 (95% CI 1.3 to 4.3), p=0.004; IMV vs COT: 2.3 (95% CI 1.3 to 4.1), p=0.005) at 6 months, but not at 12 months.

### Risk factors of recovery

**Figure 4:**
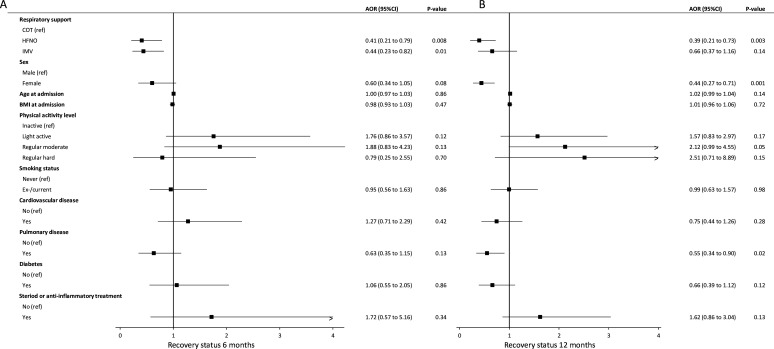
Recovery status and its risk factors at 6 and 12 months after hospital discharge. Forest plots presenting risk factors for self-reported recovery status from COVID-19. Data are obtained using multivariable generalised estimating equations analysis. Recovery status from COVID-19 was assessed with the Core Outcome Measure for Recovery. Recovery was dichotomised into good recovery (complete or mostly recovered) and poor recovery (not recovered at all, somewhat recovered, or half recovered). AOR, adjusted OR; BMI, body mass index; COT, conventional oxygen therapy; HFNO, high-flow nasal oxygen; IMV, invasive mechanical ventilation.

Poor recovery at 6 months was more likely in participants receiving HFNO or IMV compared with COT (figure 4A). At 12 months, in the multivariable analysis, the HFNO group was more likely to report poor recovery compared with the COT group. The duration of HFNO treatment was not associated with recovery at both time points. Other risk factors for poor recovery were female sex and pulmonary history (figure 4B).

### Overlapping outcomes

To understand the interplay between multidimensional outcomes, we examined the overlap between HRQoL, symptom clusters and self-reported recovery at 6 and 12 months. Patients were stratified into quartiles based on the EQ-5D-5L utility score to visualise differences in health status (online supplemental figure S3). In the lowest quartile (25th percentile), nearly all patients reported persistent symptoms across multiple clusters, and 43% experienced both symptoms in all clusters and poor recovery. In contrast, patients in the highest quartile (100th percentile) had the lowest symptom burden, with only 5% reporting poor recovery and <1% experiencing both symptoms in all clusters and poor recovery. Notably, a substantial overlap in outcomes was observed, following a gradient across health status levels.

## Discussion

In this study, we used data from two large prospective multicentre cohorts of patients hospitalised for COVID-19 to investigate the role of COVID-19 disease severity and other risk factors on long-term multidimensional endpoints. Our key findings include: (1) 6 and 12 months after hospitalisation for COVID-19, HRQoL was still impaired, symptoms persisted and self-perceived recovery was often poor, (2) COVID-19 disease severity was not associated with HRQoL, (3) patients treated with HFNO more frequently experienced respiratory symptoms and reported poorer recovery and (4) in contrast to COVID-19 disease severity, female sex, younger age and a pulmonary history were more consistent risk factors across long-term multidimensional outcomes.

Hospitalised COVID-19 patients typically require monitored oxygen therapy due to their high disease burden, increasing their susceptibility to long-term health problems compared with those not hospitalised.^6 7^ Our study provided further data supporting that hospitalised patients experience multidimensional impairments up to 12 months postdischarge,^5^ with a clear gradient and overlap between outcomes. These findings underscore the multidimensional burden of long-term outcomes and emphasise the need for a multidimensional framework when assessing recovery after hospitalisation for COVID-19. However, the relation between COVID-19 disease severity and long-term outcomes in hospitalised patients is less clear.^49^,^12 20^ Studies suggest that hospitalised patients with more severe initial disease may be at higher risk.^410^,^12^ However, COVID-19 disease severity was not a consistent risk factor across our multidimensional outcomes.^920^,^22^ Moreover, the large sample of hospitalised patients receiving various respiratory support modalities enabled us to conduct a comprehensive multivariable analysis of COVID-19 disease severity and other risk factors on long-term multidimensional outcomes. This contrasts with previous studies that were in part hampered by their single-centre design, limited sample size (especially NIV/HFNO treatment; WHO score 4),^4 11 21^ often single long-term outcome measure,^6 7 11 21^ and comparison to patients not requiring oxygen at all.^4 11^ To differentiate between disease severity, several studies dichotomised between ICU and non-ICU admission.^12 22^ The three levels of respiratory support are a more valid proxy for disease severity, as the true disease severity of ICU-admitted patients may vary considerably between centres and countries.^23^

Some notable associations between maximal level of respiratory support and outcomes were, however, observed. IMV treatment especially impacted the persistence of physical symptoms, while HFNO treatment posed a risk for persistent respiratory symptoms, particularly dyspnoea and cough, at 6 months, and fatigue and poor recovery up to 12 months. These differences in associations between 6 and 12 months may reflect a degree of recovery, fluctuations in symptom presence, or variation in long-term symptom persistence.^24^ Moreover, the duration of HFNO therapy was associated with respiratory symptoms at 6 months, but not at 12 months. However, these findings are exploratory and require confirmation. Aligning our findings, previous studies showed that patients with severe COVID-19 (HFNO, NIV or IMV therapy) experienced delayed pulmonary recovery, yet only one study differentiated between HFNO/NIV and IMV.^21 25^ Possible explanations for why patients on (prolonged) HFNO more frequently experience late respiratory symptoms remain hypothetical but may be related to the potential damaging effect of prolonged vigorous efforts during HFNO in severely hypoxaemic patients.^26^

Ideally, risk factors should be easily collected within the standard of care, guiding early identification of patients at risk for relevant outcomes. However, due to the multifaceted nature and lack of definitive and validated outcome measures, let alone a golden standard,^27^ assessing long covid is challenging. Various metrics, including symptomatology, HRQoL, pulmonary function or exercise testing, are used to ‘define’ and evaluate long covid, resulting in clinical risk factors that are variably related to different post-COVID-19 outcomes.^25 28^ Ideally, selected risk factors should either directly relate to the gold standard or align with multiple relevant endpoints recognised by stakeholders. Due to inconsistent findings and lack of gold standard diagnostics for long covid, we identified alternative risk factors with consistent associations across multiple outcomes. In multivariable GEE analyses, we observed that sex, age and pulmonary history were independent risk factors across long-term multidimensional outcomes. These risk factors have indeed surfaced repeatedly^10 17 19 21^ and could be used to guide aftercare strategies. In our cohort, we observed that a greater proportion of patients treated with IMV, indicating more severe disease, received medical or skilled nursing rehabilitation. More intensive rehabilitation might have a positive effect on long-term outcomes,^29 30^ potentially preventing even more severe long-term outcomes.

Our study indicates that COVID-19 disease severity as measured by the maximal level of respiratory support is not a consistent risk factor for long-term outcomes in hypoxaemic patients hospitalised for COVID-19. Instead, sex, age and pulmonary history are more consistently associated with multiple domains of outcomes. Given the expected large number of patients with long covid, the strain on healthcare systems could become overwhelming.^31^ Screening could help prioritise limited societal resources by directing posthospital aftercare therapy to those most in need. Factors relevant for multiple long-term outcomes may well serve as relevant screening criteria. These screening criteria can be further explored for their applicability in other postinfectious disease syndromes and the post-IC syndrome, given the evident similarities with long covid.

The major strengths of our study are its prospective multicentre design, its significant sample size covering a wide variety of in-hospital COVID-19 disease severities, and its relatively high response rate. Also, we collected a comprehensive set of long-term multidimensional patient-centred outcomes and patient-related variables enabling us to perform an in-depth analysis and adjust for a multitude of relevant confounders.

Our study also comes with several limitations. First, we collected data during the first and second pandemic waves, while the virus, treatments and patients at risk changed over time. We could partly address this by adjusting for treatment differences in our multivariable analyses. Second, non-Dutch-speaking patients were excluded due to study procedures, potentially overlooking individuals with a migration background known to be prone to impaired HRQoL recovery. Also, at 6 months, outcomes might be underestimated as non-responders showed more severe disease characteristics than responders. However, at 12 months, this difference was not observed. Given the consistent outcomes at 6 and 12 months, overall underestimation is unlikely to play a major role. Further, the participants’ pre-COVID HRQoL may well impact the evolution of HRQoL after the disease episode of interest. Although pre-COVID HRQoL data were not collected, data on physical activity prior to infection was and was included in the multivariable GEE analyses. Additionally, dichotomised scoring of symptoms may have led to missing nuances in symptom severity. By using symptom clusters, we aimed to capture broader symptom severity. Also, our GEE analysis may have suffered from not including all relevant confounders and/or risk factors, despite our efforts to include those deemed relevant at the time.

In conclusion, up to 12 months after hospitalisation for COVID-19, HRQoL remained reduced compared with general population, symptoms persisted and a substantial number of patients reported incomplete recovery. COVID-19 disease severity was not consistently associated across an array of long-term multidimensional outcomes. Patients treated with HFNO reported more respiratory symptoms and poor recovery. Female sex, younger age and pulmonary history were more consistently associated with outcomes. Our findings collectively suggest that factors other than COVID-19 disease severity are possibly better factors to guide and prioritise aftercare therapy for long-term problems.

## Supplementary material

10.1136/bmjresp-2024-002789 online supplemental file 1

## Data Availability

Data are available on reasonable request.
