# Supplementary material for: Long-term multidimensional patient-centred outcomes after hospitalisation for COVID-19: do not only focus on disease severity
Source: BMJ Open Respir Res. 2025 Jun 8;12(1):e002789. doi: 10.1136/bmjresp-2024-002789 (PMC12161316; doi:10.1136/bmjresp-2024-002789)
Supplement: online supplemental file 1 [file bmjresp-12-1-s001.pdf]

Supplemental material

Long-term multidimensional patient-centered outcomes after hospitalization for COVID-19; don’t only focus on disease severity

Authors: L. Martine Bek[1*], MSc, Yasemin Türk[2], PhD, Matthijs L. Janssen[3,4,5], MD, Gemma Weijsters[2], MD, Julia C. Berentschot[5], MSc, Rita J.G. van den Berg-Emons[1], PhD, Majanka H. Heijenbrok-Kal[1,6], PhD, Gerard M. Ribbers[1,6], PhD, Joachim G.J.V. Aerts[5], PhD, Wessel E.J.J. Hanselaar[2], PhD, Henrik Endeman[4], PhD, Merel E. Hellemons[5], PhD, Evert-Jan Wils[3,4], PhD, on behalf of the CO-FLOW collaboration[**] group and Dutch HFNO COVID-19 study group[***]

Affiliations

1. Department of Rehabilitation Medicine, Erasmus MC, University Medical Center Rotterdam, The Netherlands.
2. Department of Respiratory Medicine, Franciscus Gasthuis & Vlietland Hospital, Rotterdam, The Netherlands.
3. Department of Intensive Care, Franciscus Gasthuis & Vlietland Hospital, Rotterdam, The Netherlands.
4. Department of Intensive Care, Erasmus MC, University Medical Center Rotterdam, The Netherlands.
5. Department of Respiratory Medicine, Erasmus MC, University Medical Center Rotterdam, The Netherlands.
6. Rijndam rehabilitation, Rotterdam, The Netherlands.

, * Collaboration group and study group are mentioned on the last page

Table of Contents

STROBE Statement 3

Figure S1. Flowchart of HFNO COVID-19 study and CO-FLOW study. 5

Figure S2. Fatigue and cognitive symptom cluster and their risk factors at 6 and 12 months after hospital discharge. 6

Figure S3. Overlap between HRQoL, symptom clusters, and self-reported recovery at 6 and 12 months. 7

Table S1. Demographics and clinical characteristics collected and included as risk factors in the analyses. 9

Table S2. Demographics and clinical characteristics of non-responders and responders at 6 and 12 months. 10

Table S3. Demographics and clinical characteristics of patients at 6 months and patients at 12 months follow-up. 11

Table S4. EQ-5D-5L utility score and EQ-VAS scores at 6 and 12 months for the full cohort and split by maximal level of respiratory support. 12

Table S5. Domains of EQ-5D-5L utility score split by maximal level of respiratory support at 6 months. 13

Table S6. Domains of EQ-5D-5L utility score split by maximal level of respiratory support at 12 months. 14

Table S7. Prevalence of symptoms per symptom clusters at 6 and 12 months for the full cohort and split by maximal level of respiratory support. 15

Table S8. Self-reported recovery status at 6 and 12 months for the full cohort and split by maximal level of respiratory support. 16

STROBE Statement

	Item No	Recommendation	Page No
**Title and abstract**	1	(*a*) Indicate the study's design with a commonly used term in the title or the abstract<br>(*b*) Provide in the abstract an informative and balanced summary of what was done and what was found	1-3
**Introduction**			
Background/rationale	2	Explain the scientific background and rationale for the investigation being reported	5
Objectives	3	State specific objectives, including any prespecified hypotheses	5
**Methods**			
Study design	4	Present key elements of study design early in the paper	6
Setting	5	Describe the setting, locations, and relevant dates, including periods of recruitment, exposure, follow-up, and data collection	6
Participants	6	(*a*) Give the eligibility criteria, and the sources and methods of selection of participants. Describe methods of follow-up<br>(*b*) For matched studies, give matching criteria and number of exposed and unexposed	6
Variables	7	Clearly define all outcomes, exposures, predictors, potential confounders, and effect modifiers. Give diagnostic criteria, if applicable	7-8
Data sources/ measurement	8*	For each variable of interest, give sources of data and details of methods of assessment (measurement). Describe comparability of assessment methods if there is more than one group	6-8
Bias	9	Describe any efforts to address potential sources of bias	7-8
Study size	10	Explain how the study size was arrived at	-
Quantitative variables	11	Explain how quantitative variables were handled in the analyses. If applicable, describe which groupings were chosen and why	8
Statistical methods	12	(*a*) Describe all statistical methods, including those used to control for confounding<br>(*b*) Describe any methods used to examine subgroups and interactions<br>(*c*) Explain how missing data were addressed<br>(*d*) If applicable, explain how loss to follow-up was addressed<br>(*e*) Describe any sensitivity analyses	8
**Results**			
Participants	13*	(a) Report numbers of individuals at each stage of study—eg numbers potentially eligible, examined for eligibility, confirmed eligible, included in the study, completing follow-up, and analysed<br>(b) Give reasons for non-participation at each stage<br>(c) Consider use of a flow diagram	9-10
Descriptive data	14*	(a) Give characteristics of study participants (eg demographic, clinical, social) and information on exposures and potential confounders<br>(b) Indicate number of participants with missing data for each variable of interest<br>(c) Summarise follow-up time (eg, average and total amount)	9-10
Outcome data	15*	Report numbers of outcome events or summary measures over time	9-14

Main results	16	(*a*) Give unadjusted estimates and, if applicable, confounder-adjusted estimates and their precision (eg, 95% confidence interval). Make clear which confounders were adjusted for and why they were included<br>(*b*) Report category boundaries when continuous variables were categorized<br>(*c*) If relevant, consider translating estimates of relative risk into absolute risk for a meaningful time period	9-14
Other analyses	17	Report other analyses done—eg analyses of subgroups and interactions, and sensitivity analyses	9-14
**Discussion**			
Key results	18	Summarise key results with reference to study objectives	14,18
Limitations	19	Discuss limitations of the study, taking into account sources of potential bias or imprecision. Discuss both direction and magnitude of any potential bias	17-18
Interpretation	20	Give a cautious overall interpretation of results considering objectives, limitations, multiplicity of analyses, results from similar studies, and other relevant evidence	14-18
Generalisability	21	Discuss the generalisability (external validity) of the study results	17-18
**Other information**			
Funding	22	Give the source of funding and the role of the funders for the present study and, if applicable, for the original study on which the present article is based	22

*Give information separately for exposed and unexposed groups.

Figure S1. Flowchart of HFNO COVID-19 study and CO-FLOW study.

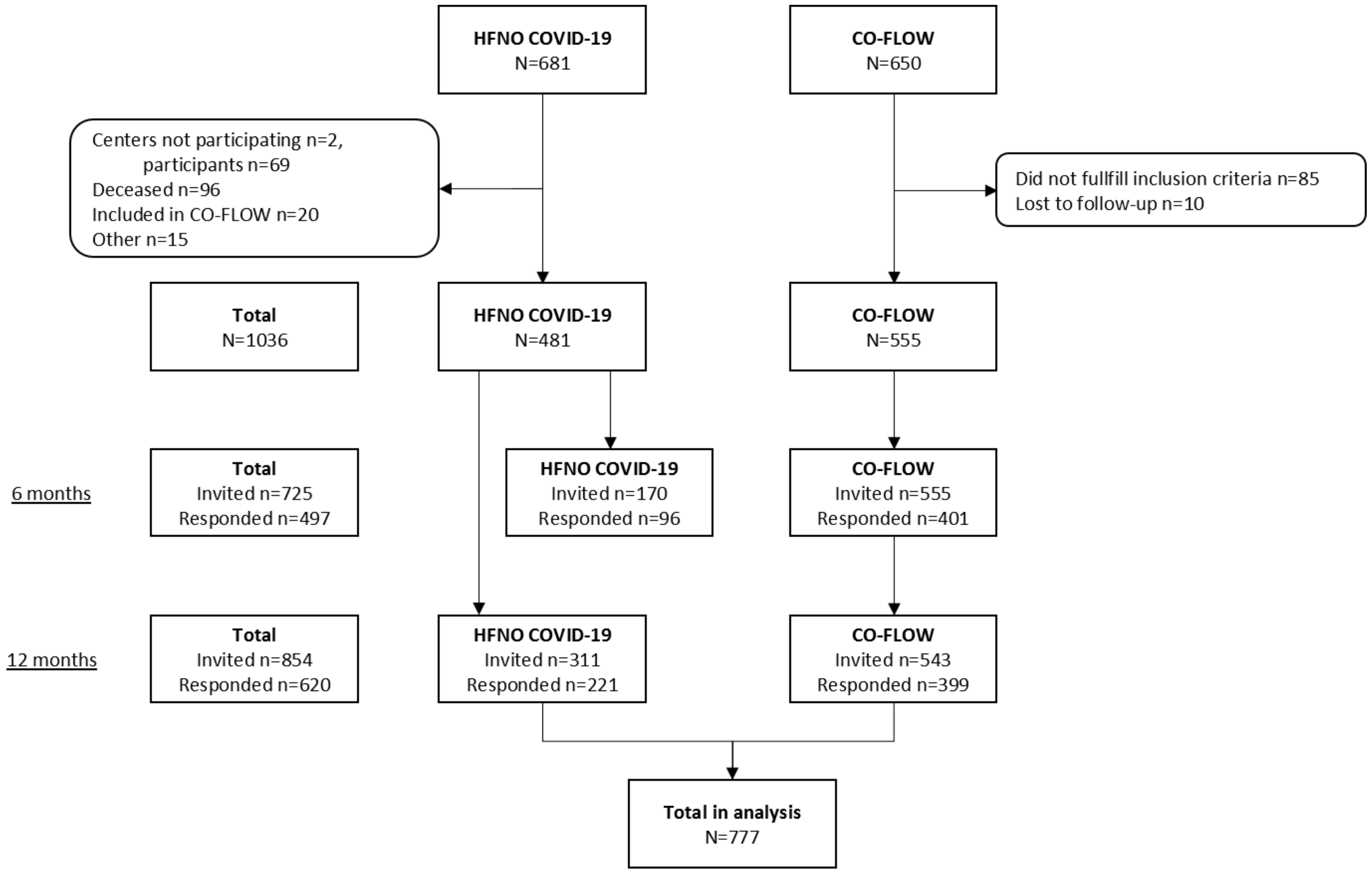

Figure S1. Flowchart of HFNO COVID-19 study and CO-FLOW study.
Of the centers participating in the HFNO COVID-19 study, two were unable to take part in this follow-up study. As a result, patients from these centers (n=69) were not invited.

Figure S2. Fatigue and cognitive symptom cluster and their risk factors at 6 and 12 months after hospital discharge.

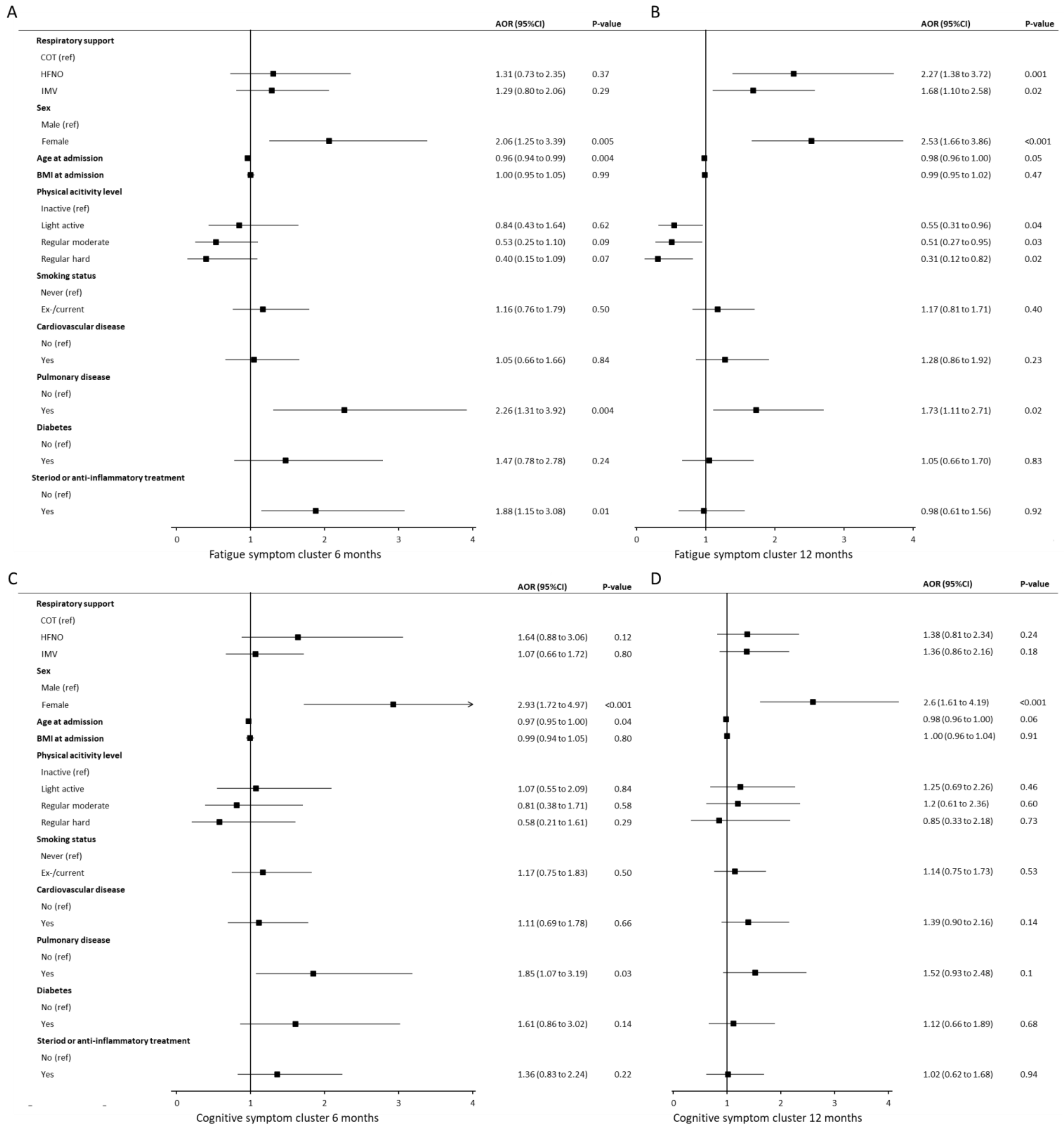

Figure S2. Fatigue and cognitive symptom cluster and their risk factors at 6 and 12 months after hospital discharge. Forest plots presenting risk factors of A: fatigue symptom cluster at 6 months, B: fatigue symptom cluster at 12 months, C: cognitive symptom cluster at 6 months, and D: cognitive symptom cluster at 12 months post-discharge. Data are obtained using multivariable binary Generalized Estimating Equations analysis. Symptoms were assessed with the Corona Symptom Checklist (24). AOR, Adjusted Odds Ratio; CI, Confidence Interval; COT, Conventional Oxygen Therapy; HFNO, High Flow Nasal Oxygen; IMV, Invasive Mechanical Ventilation; BMI, Body Mass Index.

Figure S3. Overlap between HRQoL, symptom clusters, and self-reported recovery at 6 and 12 months.

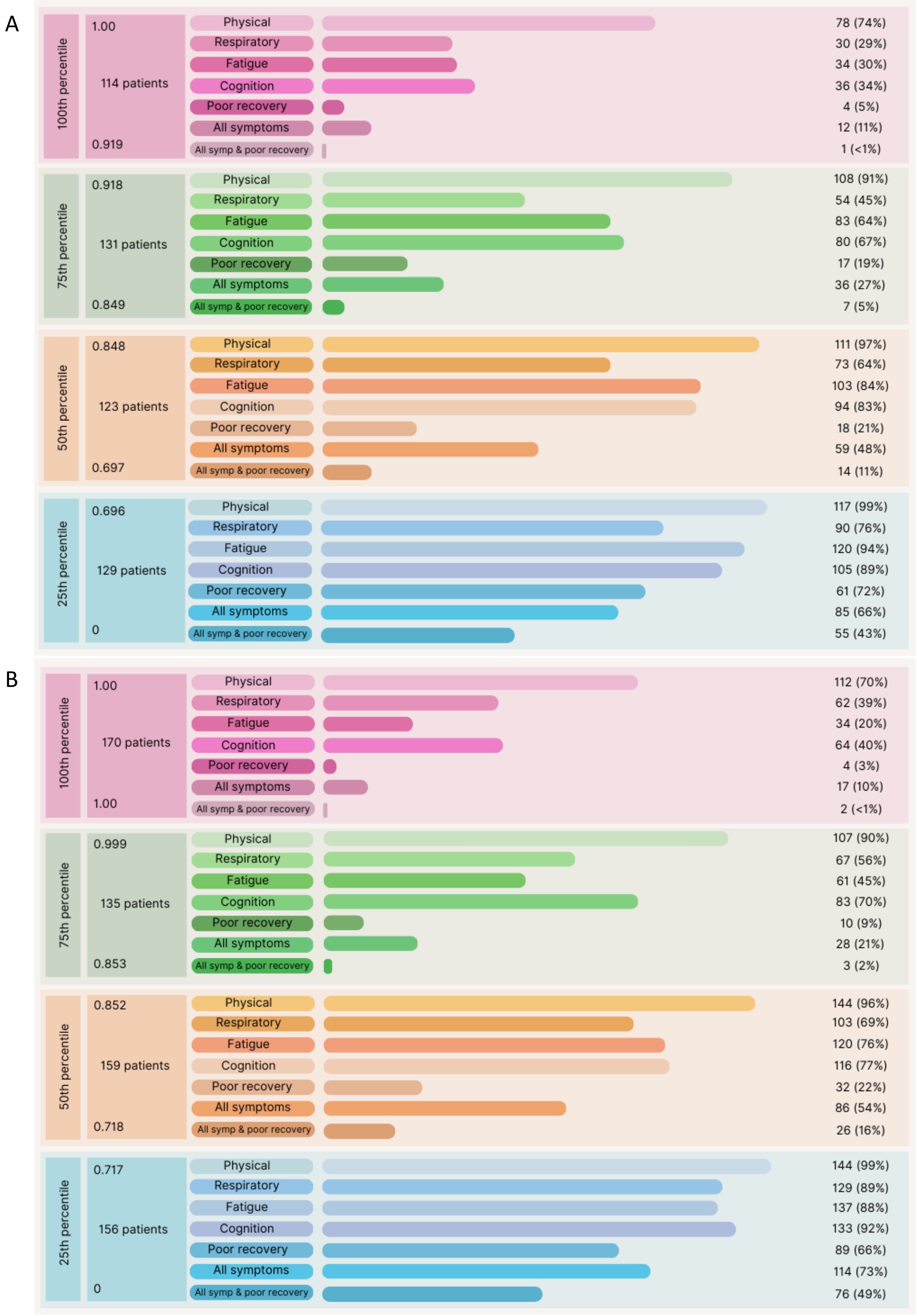

Figure S3. Overlap between HRQoL, symptom clusters, and self-reported recovery at 6 and 12 months.

The figure presents the distribution of persistent symptom clusters and self-reported poor recovery at 6 (A) and 12 (B) months post-discharge, stratified by EQ-5D-5L utility score quartiles (25th, 50th, 75th 100th percentiles). The 25th percentile represents patients with the lowest HRQoL and the 100th reflects those with the highest. For each quartile, the defining utility score and corresponding number of patients are shown on the left. Symptom and recovery data are presented per group across the following domains: physical, respiratory, fatigue, cognitive, self-reported recovery, presence of all symptoms, and the combination of all symptoms with poor recovery. The "All symptoms" bar captures patients reporting symptoms in all clusters, while “All symp & poor recovery” represents those experiencing both symptoms in all clusters and poor recovery.

Table S1. Demographics and clinical characteristics collected and included as risk factors in the analyses.

**Collected variables**	**Included as risk factor in GEE analysis**
Sex, *female/male*	X
Age at admission, *years*	X
BMI at admission, *kg/m²*	X
Physical activity level prior to COVID-19 infection, *inactive/light / moderate/vigorous* [1]	X
Smoking status, *ex-/current vs never*	X
Medical history, *cardiovascular disease, pulmonary disease diabetes; yes/no*	X
C-reactive protein levels at admission, *mg/L*	
Creatinine levels at admission, *umol/L*	
Pharmacological treatment for COVID-19 during admission, *1] Antivirals (Remdesivir, Lopinavir/Ritonavir), 2] Steroids (Dexamethasone, Prednisone, high-dose Methylprednisolone), and 3] Anti-inflammatory agents (anti-IL-6R: Tocilizumab, Sarilumab; anti-IL-1R: Anakinra)*	X[a]
Type of respiratory support, *COT/HFNO/IMV*	X
Duration of respiratory support, *days*	
ICU admission, *yes/no*	
ICU length of stay, *days*	
Hospital length of stay, *days*	

GEE; generalized estimating equations; BMI, Body Mass Index; COT, Conservative Oxygen Therapy; HFNO, High Flow Nasal Oxygen; IMV, Invasive Mechanical Ventilation; ICU, Intensive Care Unit

[a]Pharmacological treatment was analyzed as steroids and/or anti-inflammatory agents (yes/no).

1. Grimby G, Börjesson M, Jonsdottir IH, Schnohr P, Thelle DS, Saltin B. The "Saltin-Grimby Physical Activity Level Scale" and its application to health research. Scand J Med Sci Sports. 2015;25 Suppl 4:119-25.

Table S2. Demographics and clinical characteristics of non-responders and responders at 6 and 12 months.

	Non-responders 6M	Responders 6M	P value[a]	Non-responders 12M	Responders 12M	P value[a]
N	228	497		234	620	
**Demographics**						
Sex, *female*	69 (30.3)	155 (31.2)	0.80	61 (26.1)	185 (29.8)	0.28
Age at admission, median (IQR), *years*	60.0 (52.0-67.0)	60.0 (54.0-67.5)	0.25	61.0 (52.0-68.0)	61.0 (54.0-68.0)	0.30
BMI at admission, median (IQR), *kg/m²*	29.4 (26.1-33.7)	28.1 (25.6-31.9)	0.007	29.3 (26.1-33.2)	28.6 (25.9-32.3)	0.13
Physical activity level			0.76			0.48
Inactive	19 (13.5)	70 (14.2)		18 (13.2)	90 (14.7)	
Light	81 (57.4)	258 (52.4)		76 (55.9)	325 (53.0)	
Moderate	34 (24.1)	135 (27.4)		32 (23.5)	169 (27.6)	
Vigorous	7 (5.0)	29 (5.9)		10 (7.4)	29 (4.7)	
Smoking status, *ex-/current vs. never*	84 (41.8)	269 (56.0)	<0.001	100 (49.8)	289 (50.8)	0.80
**Clinical characteristics**						
Medical history						
≥1	180 (87.8)	388 (83.6)	0.10	192 (88.5)	495 (86.7)	0.50
Obesity (BMI≥30)	101 (46.5)	182 (37.3)	0.02	103 (45.4)	238 (39.9)	0.15
Cardiovascular disease	93 (40.8)	180 (36.2)	0.24	103 (44.0)	245 (39.5)	0.23
Pulmonary disease	51 (22.4)	117 (23.5)	0.73	50 (21.4)	146 (23.5)	0.50
Diabetes	61 (26.8)	82 (16.5)	0.001	70 (29.9)	126 (20.3)	0.003
Respiratory support			0.004			0.95
COT	60 (28.3)	203 (40.8)		68 (30.9)	191 (30.8)	
HFNO	58 (27.4)	216 (25.4)		72 (32.7)	197 (31.8)	
IMV	94 (44.3)	168 (33.8)		80 (36.4)	232 (37.4)	
Steroids or anti-inflammatory treatment	173 (75.9)	108 (82.1)	0.05	206 (88.0)	509 (82.1)	0.04
ICU admission	131 (57.5)	213 (42.9)	<0.001	126 (53.8)	306 (49.4)	0.24
Hospital LOS, median (IQR), *days*	16.0 (8.0-35.0)	13.0 (7.0-25.0)	0.004	13.0 (7.0-31.0)	14.0 (8.0-27.0)	0.85

Data are presented as median (interquartile range), or n (%). P value is obtained using Kruskal-Wallis test, or Chi-squared test as appropriate. [a] P value indicates the difference in baseline characteristics between responders and non-responders at 6 and 12 months. M, months; BMI, Body Mass Index; COT, Conventional Oxygen Therapy; HFNO, High Flow Nasal Oxygen; IMV, Invasive Mechanical Ventilation; ICU, Intensive Care Unit; LOS, Length of Stay.

Table S3. Demographics and clinical characteristics of patients at 6 months and patients at 12 months follow-up.

	6 Months	12 Months	P value
N	497	620	
**Demographics**			
Sex, *female*	155 (31.2)	185 (29.8)	0.29
Age at admission, median (IQR), *years*	60.0 (54.0-67.5)	61.0 (54.0-68.0)	0.14
BMI at admission, median (IQR), *kg/m^2*	28.1 (25.6-31.9)	28.6 (25.9-32.3)	0.12
Physical activity level			0.63
Inactive	70 (14.2)	90 (14.7)	
Light	258 (52.4)	325 (53.0)	
Moderate	135 (27.4)	169 (27.6)	
Vigorous	29 (5.9)	29 (4.7)	
Smoking status, *ex-/current vs. never*	269 (56.0)	289 (50.8)	0.11
**Clinical characteristics**			
Medical history			
≥1	388 (83.6)	495 (86.7)	0.17
Obesity (BMI≥30)	182 (37.3)	238 (39.9)	0.25
Cardiovascular disease	180 (36.2)	245 (39.5)	0.19
Pulmonary disease	117 (23.5)	146 (23.5)	0.98
Diabetes	82 (16.5)	126 (20.3)	0.12
Respiratory support			<0.001
COT	203 (40.8)	191 (30.8)	
HFNO	126 (25.4)	197 (31.8)	
IMV	168 (33.8)	232 (37.4)	
Steroids or anti-inflammatory treatment	408 (82.1)	509 (82.1)	0.05
ICU admission	213 (42.9)	306 (49.4)	0.01
Hospital LOS, median (IQR), *days*	13.0 (7.0-25.0)	14.0 (8.0-27.0)	0.02

Data are presented as median (interquartile range), or n (%). P value is obtained using Kruskal-Wallis test, or Chi-squared test as appropriate. BMI, Body Mass Index; COT, Conventional Oxygen Therapy; HFNO, High Flow Nasal Oxygen; IMV, Invasive Mechanical Ventilation; ICU, Intensive Care Unit; LOS, Length of Stay.

Table S4. EQ-5D-5L utility score and EQ-VAS scores at 6 and 12 months for the full cohort and split by maximal level of respiratory support.

	**Dutch norm [a]**	**Full cohort**	**COT**	**HFNC**	**IMV**	**P value [c]**
**6 Months**						
N		497	203 (40.8)	126 (25.4)	168 (33.8)	
**EQ-5D-5L utility score [b]**						0.17
Mean (SD)	0.87 (0.17)	0.78 (0.22)	0.80 (0.21)	0.76 (0.22)	0.77 (0.85)	
Median (IQR)	0.89 (0.82-1.00)	0.85 (0.70-0.92)	0.85 (0.72-1.00)	00.81 (0.67-0.90)	0.85 (0.70 -0.92)	
**EQ-VAS**						0.47
Mean (SD)	80.6 (14.7)	73.1 (17.3)	73.9 (18.3)	71.6 (15.8)	73.1 (17.3)	
Median (IQR)	81.0 (72.0-90.0)	75.0 (61.0-86.5)	76.0 (65.0-90.0)	73.5 (60.0-81.8)	75.0 (60.0-87.8)	
**12 Months**	**Dutch norm [a]**	**Full cohort**	**COT**	**HFNC**	**IMV**	**P value [c]**
N		620	191 (30.8)	197 (31.8)	232 (37.4)	
**EQ-5D-5L utility score**						0.11
Mean (SD)	0.87 (0.17)	0.80 (0.22)	0.82 (0.20)	0.81 (0.22)	0.77 (0.24)	
Median (IQR)	0.89 (0.82-1.00)	0.85 (0.72-1.00)	0.88 (0.75-1.00)	0.88 (0.74-1.00)	0.82 (0.70-1.00)	
**EQ-VAS**						0.67
Mean (SD)	80.6 (14.7)	73.5 (17.4)	74.4 (17.3)	73.0 (16.8)	73.1 (18.1)	
Median (IQR)	81.0 (72.0-90.0)	75.0 (63.0-85.0)	80.0 (63.0-86.0)	75.0 (65.0-85.0)	75.0 (60.0-87.5)	

Data are presented as mean (standard deviation) and median (interquartile range). COT, Conventional Oxygen Therapy; HFNO, High Flow Nasal Oxygen; IMV, Invasive Mechanical Ventilation.

[a] mean (SD) Dutch norm for EQ-5D-5L utility score is 0.87 (0.17) and median (IQR) is 0.89 (0.82-1.00); mean (SD) for EQ-VAS is 80.6 (14.7) and median (IQR) is 81.0 (72.0-90.0) [16].

[b] mean (SD) and median (IQR) were obtained using descriptive statistics.

[c] P value is obtained with post-hoc analyses of univariable linear generalized estimating equations analysis.

Table S5. Domains of EQ-5D-5L utility score split by maximal level of respiratory support at 6 months.

	COT	HFNO	IMV	P value
Mobility				0.08
No	130 (64)	70 (56)	86 (51)	
Slight	30 (15)	29 (23)	41 (25)	
Moderate	32 (16)	19 (15)	25 (15)	
Severe	11 (5)	7 (6)	14 (8)	
Extreme	0 (0)	1 (1)	2 (1)	
Self-care				0.03
No	191 (94)	113 (90)	144 (86)	
Slight	9 (4)	9 (6)	15 (9)	
Moderate	2 (1)	3 (2)	8 (5)	
Severe	1 (1)	1 (1)	1 (1)	
Extreme	0 (0)	1 (1)	0 (0)	
Usual activities				0.09
No	109 (54)	52 (41)	88 (52)	
Slight	47 (23)	38 (30)	34 (20)	
Moderate	36 (18)	21 (17)	35 (21)	
Severe	10 (5)	13 (10)	8 (5)	
Extreme	1 (1)	2 (2)	3 (2)	
Pain/discomfort				0.66
No	81 (40)	49 (39)	63 (38)	
Slight	70 (35)	40 (32)	54 (32)	
Moderate	37 (18)	26 (21)	32 (19)	
Severe	13 (6)	11 (9)	19 (11)	
Extreme	2 (1)	0 (0)	0 (0)	
Anxiety/depression				0.05
No	140 (69)	71 (56)	116 (69)	
Slight	42 (21)	39 (31)	36 (21)	
Moderate	15 (7)	12 (10)	13 (8)	
Severe	5 (3)	2 (2)	3 (2)	
Extreme	1 (1)	2 (2)	0 (0)	

Data are presented as n (%). P value is obtained using Kruskal-Wallis test. COT, Conventional Oxygen Therapy; HFNO, High Flow Nasal Oxygen; IMV, Invasive Mechanical Ventilation.

Table S6. Domains of EQ-5D-5L utility score split by maximal level of respiratory support at 12 months.

	COT	HFNO	IMV	P value
Mobility				0.05
No	123 (64)	120 (61)	125 (54)	
Slight	11 (16)	40 (20)	41 (18)	
Moderate	32 (12)	27 (14)	46 (20)	
Severe	13 (7)	7 (4)	20 (9)	
Extreme	1 (1)	3 (2)	0 (0)	
Self-care				0.06
No	177 (92)	175 (89)	198 (84)	
Slight	11 (6)	12 (6)	22 (10)	
Moderate	1 (1)	6 (3)	9 (4)	
Severe	2 (1)	1 (1)	2 (1)	
Extreme	0 (0)	3 (2)	1 (1)	
Usual activities				0.06
No	116 (60)	113 (57)	115 (49)	
Slight	37 (20)	43 (22)	55 (23)	
Moderate	26 (14)	26 (13)	45 (20)	
Severe	12 (6)	13 (7)	15 (7)	
Extreme	0 (0)	2 (1)	2 (1)	
Pain/discomfort				0.07
No	85 (44)	95 (48)	89 (38)	
Slight	57 (30)	59 (30)	74 (32)	
Moderate	38 (20)	34 (17)	51 (22)	
Severe	9 (5)	8 (4)	17 (7)	
Extreme	2 (1)	1 (1)	1 (1)	
Anxiety/depression				0.67
No	133 (69)	139 (71)	157 (68)	
Slight	42 (22)	35 (18)	42 (18)	
Moderate	13 (7)	17 (9)	24 (10)	
Severe	3 (2)	5 (3)	7 (3)	
Extreme	0 (0)	1 (1)	2 (1)	

Data are presented as n (%). P value is obtained using Kruskal-Wallis test. COT, Conventional Oxygen Therapy; HFNO, High Flow Nasal Oxygen; IMV, Invasive Mechanical Ventilation.

Table S7. Prevalence of symptoms per symptom clusters at 6 and 12 months for the full cohort and split by maximal level of respiratory support.

	**Full cohort**	**COT**	**HFNO**	**IMV**
**6 Months**				
N	500	194	124	182
Physical symptoms	455 (91)	173 (89)	112 (90)	170 (93)
Respiratory symptoms	261 (52)	88 (45)	85 (69)	88 (48)
Fatigue symptoms	361 (69)	137 (64)	97 (76)	127 (69)
Cognitive symptoms	346 (69)	132 (68)	96 (77)	118 (65)
**12 Months**				
N	610	186	193	231
Physical symptoms	535 (88)	153 (82)	169 (88)	213 (92)
Respiratory symptoms	384 (63)	107 (58)	127 (66)	150 (65)
Fatigue symptoms	372 (56)	104 (49)	130 (64)	138 (57)
Cognitive symptoms	419 (69)	125 (68)	132 (68)	162 (70)

Data are presented as n (%). COT, Conventional Oxygen Therapy; HFNO, High Flow Nasal Oxygen; IMV, Invasive Mechanical Ventilation. N, Number.

Table S8. Self-reported recovery status at 6 and 12 months for the full cohort and split by maximal level of respiratory support.

	Full cohort		COT		HFNO		IMV	
**Recovery status**	**6M (n=362)**	**12M (n=570)**	**6M (n=128)**	**12M (n=162)**	**6M (n=116)**	**12M (n=188)**	**6M (n=118)**	**12M (n=220)**
Not recovered	5 (1)	3 (1)	0 (0)	0 (0)	3 (3)	2 (1)	2 (2)	1 (1)
Somewhat recovered	23 (6)	41 (7)	7 (5)	8 (5)	10 (9)	18 (10)	6 (5)	15 (7)
Half recovered	78 (22)	94 (17)	17 (13)	21 (13)	28 (24)	33 (17)	33 (28)	40 (18)
Mostly recovered	199 (55)	302 (53)	70 (55)	87 (54)	60 (52)	92 (49)	69 (58)	123 (56)
Completely recovered	57 (16)	130 (23)	34 (27)	46 (28)	15 (13)	43 (23)	8 (7)	41 (18)

Data are presented as n (%). COT, Conventional Oxygen Therapy; HFNO, High Flow Nasal Oxygen; IMV, Invasive Mechanical Ventilation; M, Months.
